# All-metal terahertz metamaterial biosensor for protein detection

**DOI:** 10.1186/s11671-021-03566-3

**Published:** 2021-06-30

**Authors:** Gangqi Wang, Fengjie Zhu, Tingting Lang, Jianjun Liu, Zhi Hong, Jianyuan Qin

**Affiliations:** 1 Institute of Optoelectronic Technology, China Jiliang University, Hangzhou, 310018 China; 2 Zhejiang Chinese Medical University, Hangzhou, China; 3 Centre for THz Research, China Jiliang University, Hangzhou, 310018 China; 4 Lab of Terahertz Photonics, China Jiliang University, Hangzhou, 310018 China

**Keywords:** Bovine serum albumin, All-metal metamaterials, Terahertz, Biosensors

## Abstract

In this paper, a terahertz (THz) biosensor based on all-metal metamaterial is theoretically investigated and experimentally verified. This THz metamaterial biosensor uses stainless steel materials that are manufactured via laser-drilling technology. The simulation results show that the maximum refractive index sensitivity and the figure of merit of this metamaterial sensor are 294.95 GHz/RIU and 4.03, respectively. Then, bovine serum albumin was chosen as the detection substance to assess this biosensor’s effectiveness. The experiment results show that the detection sensitivity is 72.81 GHz/(ng/mm^2^) and the limit of detection is 0.035 mg/mL. This THz metamaterial biosensor is simple, cost-effective, easy to fabricate, and has great potential in various biosensing applications.

## Introduction

Nowadays, doctors usually collect serum from patients in the hospital for various examinations. Such as tumor detection [1–3] and virus detection [4–6] etc. The preliminary method for tumor detection is protein detection, because proteins are the components of many tumor markers, and these biomolecules with a lot of information exist in the serum. Serum albumins are the most abundant (52–62%) total water-soluble fraction proteins in the blood plasma [7, 8]. Furthermore, nutritional and physiological functions of serum albumins make them as essential bio-macromolecules. As one of the most popular serum albumins, bovine serum albumin (BSA) is used in a lot of research fields. Therefore, the research on the detection of BSA is quite important. There are many methods for detecting and determining the concentration of BSA, including electrochemical impedance spectroscopy [9], capillary electrophoresis [10], and light scattering techniques [11]. But they all have some disadvantages, such as complicated procedures, poor reproducibility or time-consuming. Therefore, developing new biosensors is of considerable significance and quite in demand.

The THz wave is between the microwave and infrared optical wave and in the transition region from electronics to photonics. Compared with light waves, the energy of photons in this band is very low. This means that terahertz waves will not cause radiation ionization damage to biological molecules. Many biological macromolecules also have unique fingerprints in the terahertz band [12–15]. Therefore, terahertz waves have received considerable attention in the field of biosensing [16, 17].

Metamaterials are artificial electromagnetic materials composed of subwavelength structures. Their unique electromagnetic resonance has many characteristics, such as a negative RI [18, 19], electromagnetically induced transparency [20, 21], and extreme environmental sensitivity [22, 23]. Metamaterials sensitive to the surrounding environment, especially those composed of subwavelength metal structures [24–26], have been widely used to detect various biomolecules. The combination of terahertz waves and metamaterials provides a new detection method for the biomedical molecules, which cannot only achieve label-free detection, but also refresh the resolution limit of existing sensors. In addition, detection can be completed simply and rapidly using a small amount of analyte with no chemical reagents.

At THz frequencies, metamaterial production usually relies on micron-level processing methods. Photolithography [27] or electron beam lithography [28] is mainly used to transfer micro-nano patterns from the photoresistor to the surface of the functional materials, and then wet [29, 30] or dry etching [31, 32] is required to complete the final processing of the metamaterials. Through the above steps, the construction of fine graphics can be achieved, but unfortunately, most of these methods demand expensive processing equipment, high standard operation environments, and cumbersome processing procedures. Laser drilling [33, 34] is the first practical laser processing technology, and it is also one of the main application fields of laser processing. The laser beam is highly concentrated in space and time. By focusing with a lens, the spot diameter can be reduced to micron level, and the laser power density of 10^5^–10^15^ W/cm^2^ can be obtained. With such a high-power density, laser drilling can be carried out in almost any material. To the best of our knowledge, this is the first time to apply laser-drilling technology in the fabrication of metamaterial biosensors, which can significantly reduce the processing cost of metamaterial biosensors and promote their practical applications.

In this study, a highly sensitive terahertz biosensor for protein detections based on all-metal metamaterial was proposed, theoretically simulated and experimentally demonstrated. The device was simple to manufacture, cost-effective and quite stable. It was composed of stainless-steel material and manufactured using laser-drilling technology. At first, this metamaterial sensor was simulated and analyzed using the finite integral method, and the refractive index sensitivity was calculated. Then this THz metamaterial biosensor was fabricated and measured. The experiments confirmed the high sensitivity of this senor to external environment. BSA was chosen as the detection substance to assess the biosensor’s effectiveness. The Hill formula was used to fit the experimental data. A detection sensitivity of 72.81 GHz/(ng/mm^2^) and the limit of detection (LOD) of 0.035 mg/mL were obtained. The measurements were repeated three times to verify the biosensor’s reliability.

## Design

**Fig. 1:**
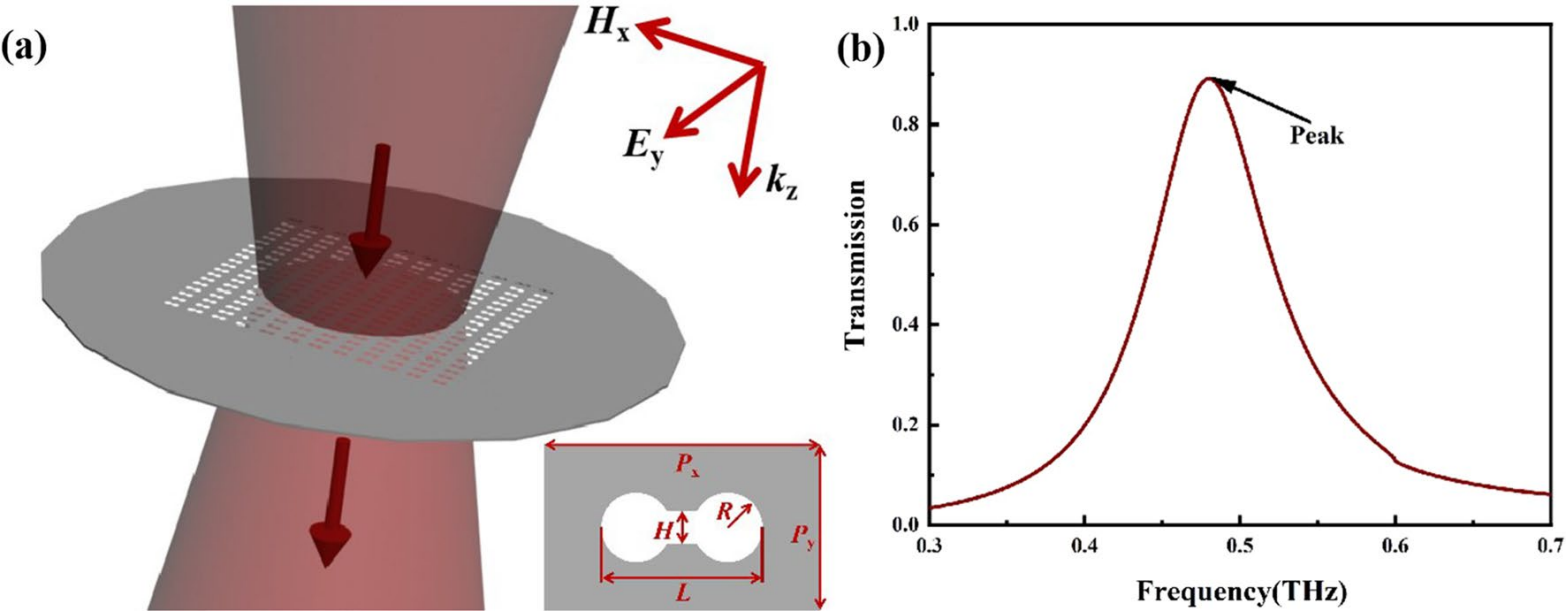
**a** Three-dimensional array diagram and cell structure diagram of the biosensor. The structural parameters are *P*_x_ = 500 μm, *P*_y_ = 300 μm, *L* = 294 μm, *H* = 60 μm, *R* = 60 μm. **b** Simulated transmission of the biosensor

Figure 1a shows the structure of the proposed all-metal metamaterial terahertz biosensor. A hollow dumbbell pattern with a periodic arrangement along the x and y directions was formed on a 50 μm thick stainless-steel plate (conductivity of 1.4 × 10^6^ S/m). The period sizes *P*_x_ and *P*_y_ of the unit structure are 500 μm and 300 μm, respectively. The hollow dumbbell’s length *L* and gap *H* are 294 μm and 60 μm, respectively. The radius *R* of circles at both ends of the hollow dumbbell is 60 μm. The biosensor had an all-metal structural design and no traditional dielectric substrate. The terahertz wave is perpendicular incident to the surface of the metamaterial biosensor.

## Methods and simulation

Then the three-dimensional full wave electromagnetic field simulation via the finite integral method (Commercial software CST) was used for the following simulations. Periodic boundary conditions were applied in the *x* and *y* directions, and the perfectly matched layer was used in the wave propagation direction of *z*. As shown in the upper right corner of Fig. 1a, the wave vector of the incident electromagnetic field *k*_z_ was a plane wave propagating in the *z*-axis, and the electric and magnetic fields were polarized along the *y*-axis and *x*-axis, respectively. As shown in Fig. 1b, there was a transmission peak at 0.48 THz.

**Fig. 2:**
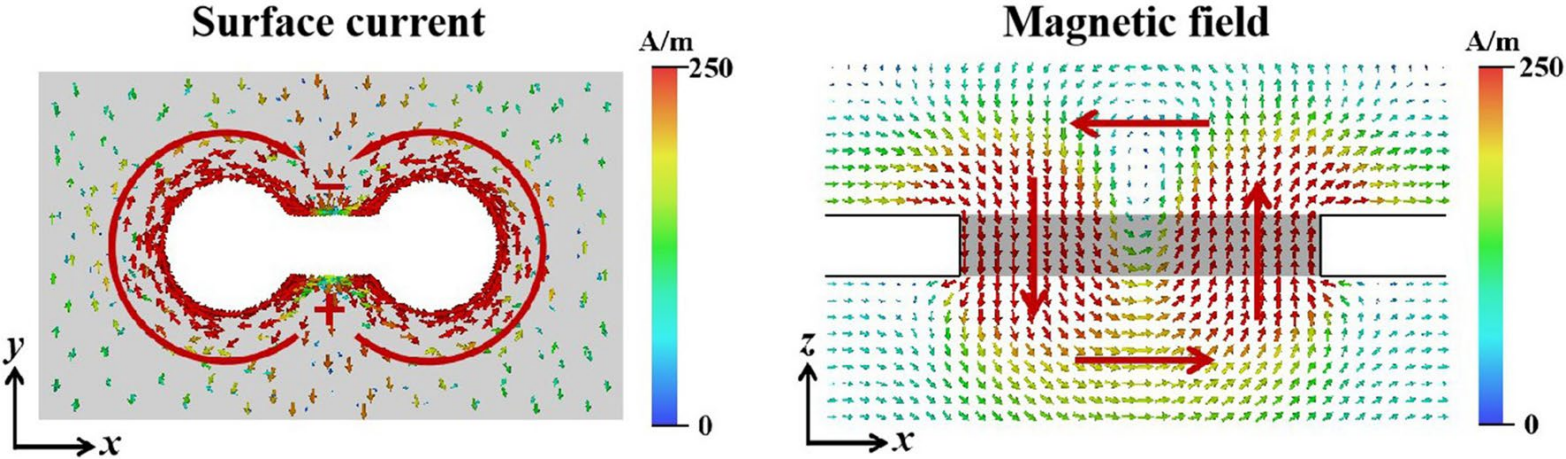
Simulated surface current distribution diagram and magnetic distribution diagram (y = 0 μm) at 0.48 THz

To study the physical mechanism of this resonance peak’s generation, the biosensor’s surface current and magnetic field at the resonance peak frequency were simulated. As shown on the left side of Fig. 2, the incident electromagnetic waves were polarized along the *y* axis, inducing charge oscillations at both ends of the opening, resulting in an electric dipole. The charge oscillations were accompanied by counter-rotating current oscillations along the rims of the two circular holes that comprised the apertures. This led to a pair of counter-oriented out-of-plane magnetic dipoles. As shown on the right side of Fig. 2, there was a pair of obvious opposite magnetic dipoles in the *z* axis connected end to end to form a toroidal dipole. Therefore, the metamaterial’s response was dominated by a combination of electric and toroidal dipoles.

**Fig. 3:**
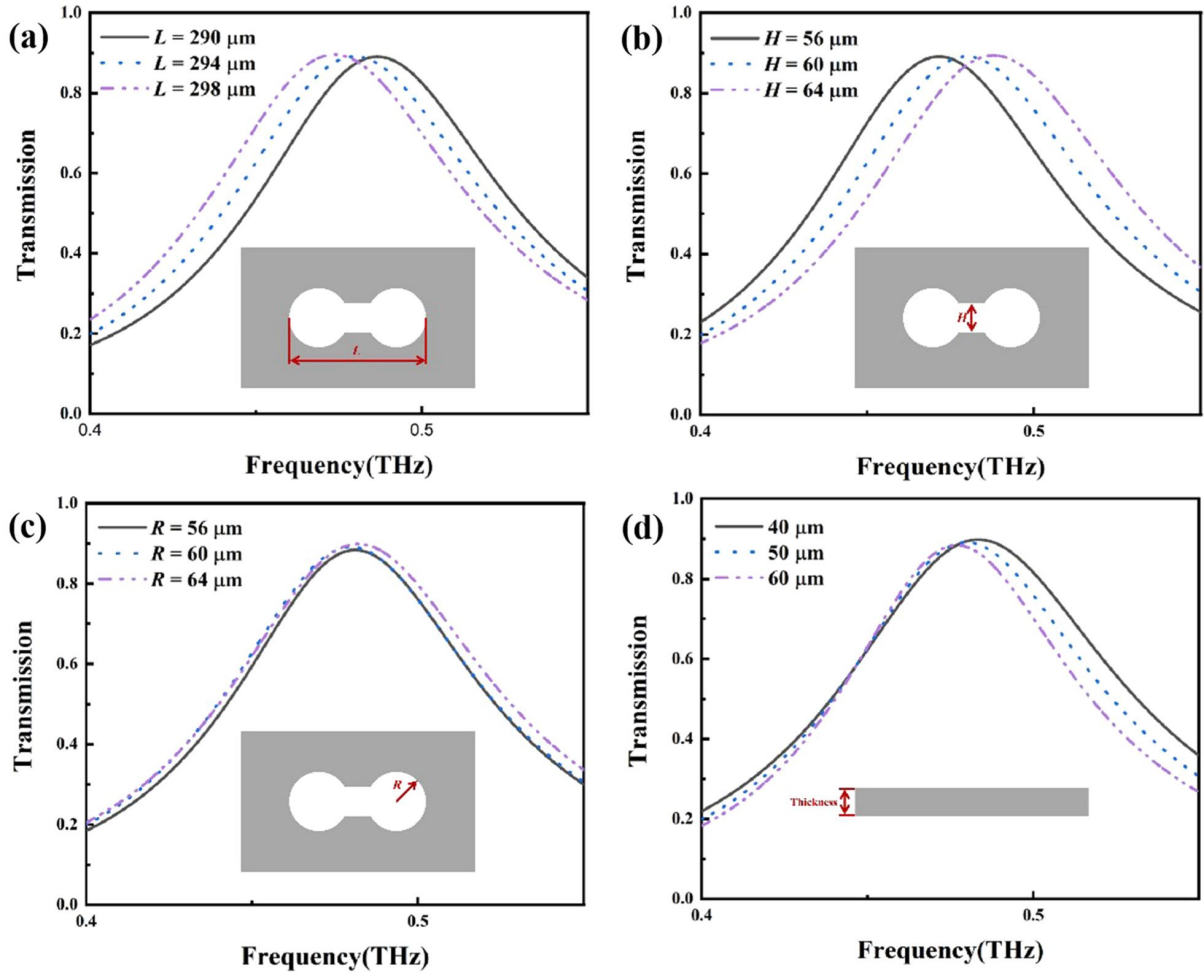
Transmission spectra of **a** different length *L*, **b** gap *H*, **c** radius *R*, and **d** stainless steel plate thickness

Because the performances of the sensor are affected by the structure parameters, it is necessary to optimize the structural parameters during the design procedure. Figure 3 shows the effect of the structural size changes on the transmission spectra. As shown in Fig. 3a, when the length of the hollow dumbbell increased from 290 to 298 μm, the peak frequency of the transmission spectra red-shifted from 0.48 THz. As shown in Fig. 3b, when the gap of the hollow dumbbell increased from 56 to 64 μm, the peak frequency of the transmission spectra blue-shifted from 0.48 THz. As *L* and *H* increased, the resonance peak started to move toward the low frequency and high frequency, respectively. When the circle’s radius varied from 56 to 64 μm and the thickness of the stainless-steel varied from 40 to 60 μm, the position of the resonance peak slightly changed. Therefore, it is easier to adjust the resonant frequency of the metamaterial biosensor by adjusting the length of the hollow dumbbell *L* and the gap of the hollow dumbbell *H.*

**Fig. 4:**
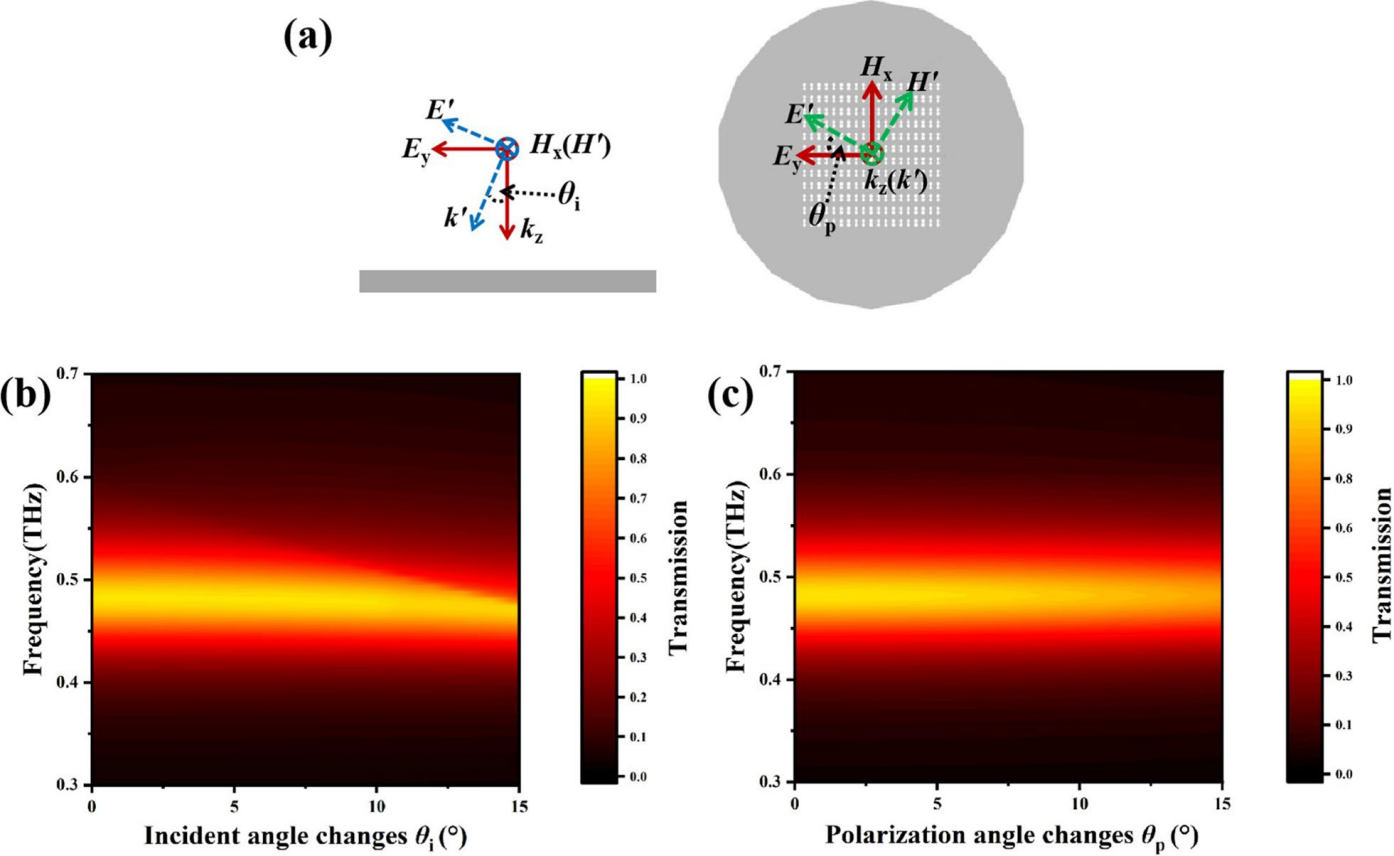
**a** Schematic diagram of changing incident angle *θ*_i_ and polarization angle *θ*_p_. Transmission spectra versus **b** the incident angle and **c** the polarization angle

It is also very important to study the influence of the incident angle and the polarization angle on the transmission spectra. The electromagnetic wave was vertically incident, and then the incident and polarization angles were changed. The definition of these angles is shown in Fig. 4a. The incident angle means *θ*_i_ in the y–z plane, and the polarization is *θ*_p_ in the x–y plane. As shown in Fig. 4b, when the incident angle changed from 0° to 15°, the difference of the resonance peak’s frequency was only 9 GHz. As shown in Fig. 4c, when the polarization angle increased from 0° to 15°, the difference in resonance peak frequency was almost 0 GHz, but the resonance peak amplitude decreased by about 0.1. This showed that the biosensor was almost insensitive to changes in the incident and polarization angles, which is beneficial for practical biosensor applications.

**Fig. 5:**
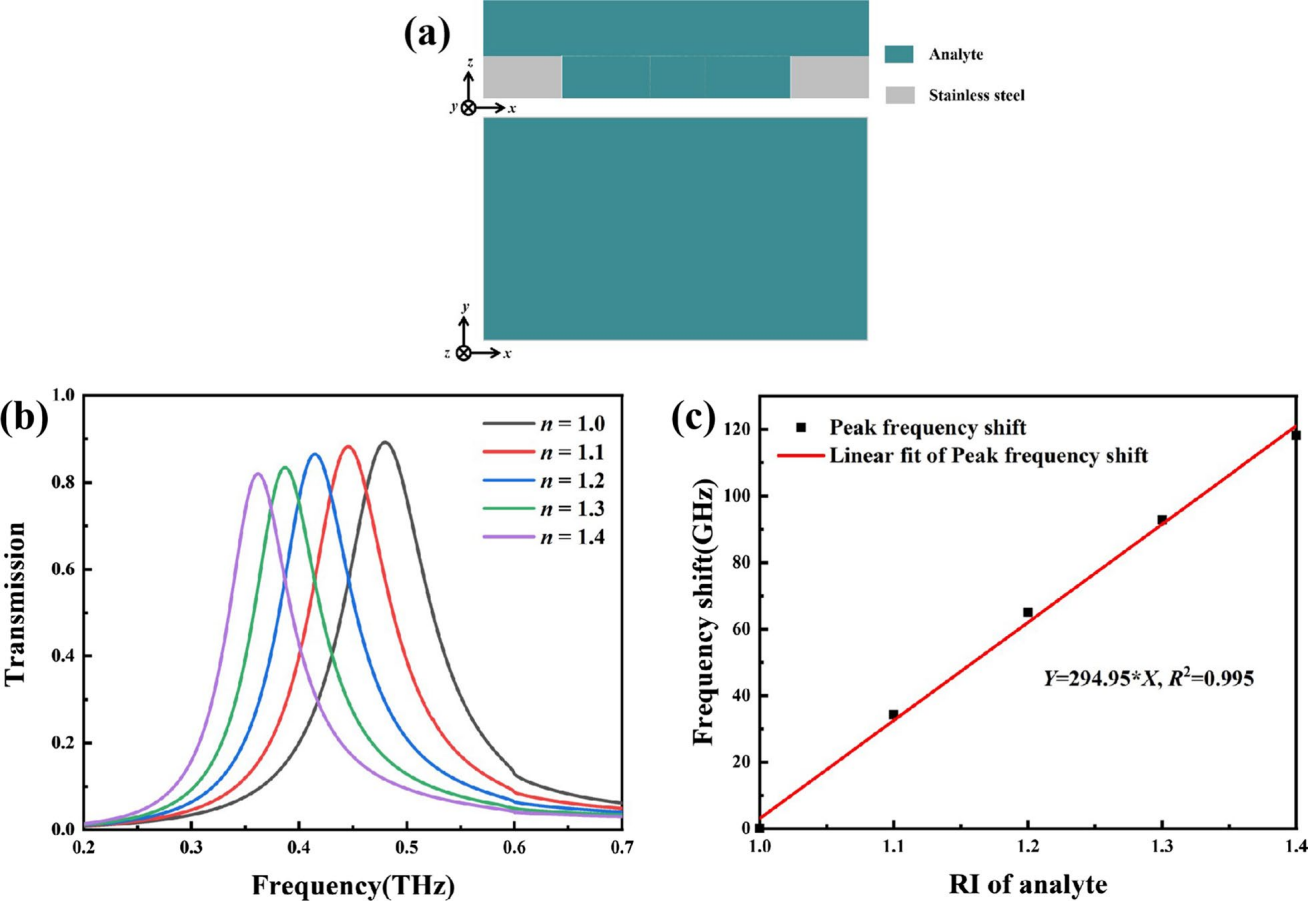
**a** Cross-sectional view and top view of the metamaterial biosensor model diagram with a 120-μm thin layer of analyte. **b** Influence of the changes in the analyte’s RI on the biosensor’s transmission spectra. **c** Corresponding linear fit of the peak’s frequency shift with the corresponding RI

To explore the biosensor’s sensing performance, a 120-μm thin layer of analyte was added to this metamaterial biosensor as shown in Fig. 5a, then different transmission spectra of this metamaterial biosensor was simulated when the refractive index of the analyte changed as shown in Fig. 5b. The RI sensitivity *S* was defined as the ratio of the variations in the transmission peak position to the RI unit (*S* = Δ*f*/Δ*n*). As the analyte’s RI increased, the resonance peak frequency red-shifted. Then the resonance peak frequency shift corresponding to each RI was collected. Good linearity was observed. The fitting result in Fig. 5c shows that the sensitivity to the RI was 294.95 GHz/RIU.

The sensing performance was also quantified using the figure of merit (FOM), which was defined as:

1 $${\text{FOM}} = \frac{S}{{{\text{FWHM}}}}$$

where *S* is the sensitivity and FHWM is the full width at half maximum of the resonance peak. The FOM of this biosensor was 4.03.

**Fig. 6:**
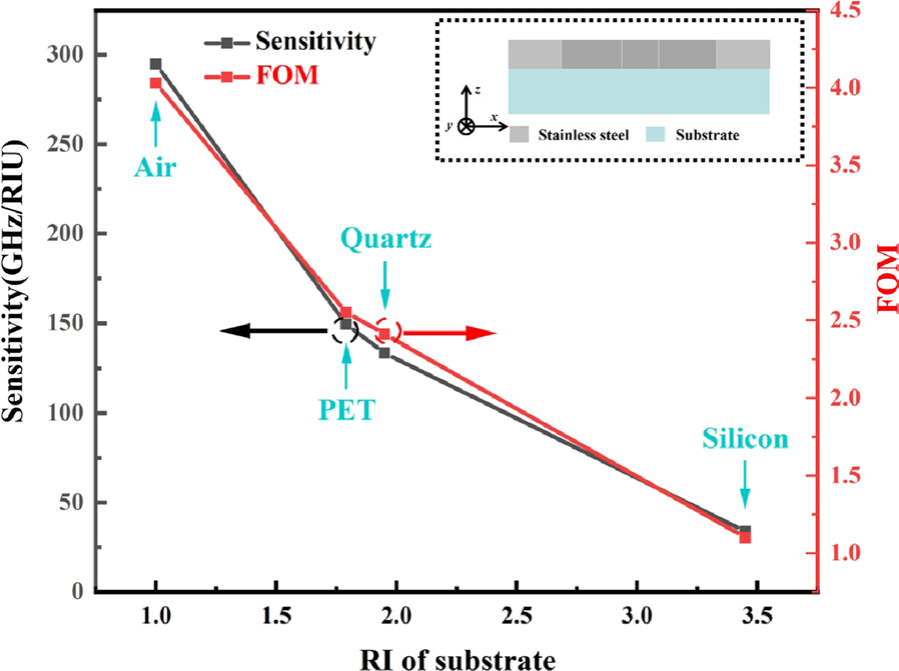
The sensitivities and FOMs when different substrates are used

For most metamaterial structures, they usually use dielectric materials as the substrates. However, this metamaterial biosensor proposed in this paper was based on an all-metal metamaterial with an all stainless-steel design and the air was used as the substrate. Compared with traditional dielectric materials, such as polyethylene terephthalate (PET), quartz, and silicon, the air has the lowest RI. To evaluate the role of the substrate, these metamaterial biosensors were simulated again using different substrates, and the refractive index sensitivities and FOM values were calculated subsequently. As shown in Fig. 6, as the RI of the substrate increased, the sensor’s RI sensitivity and FOM began to decrease. This result indicated that the biosensor with a lower substrate RI had a better sensing performance.

**Fig. 7:**
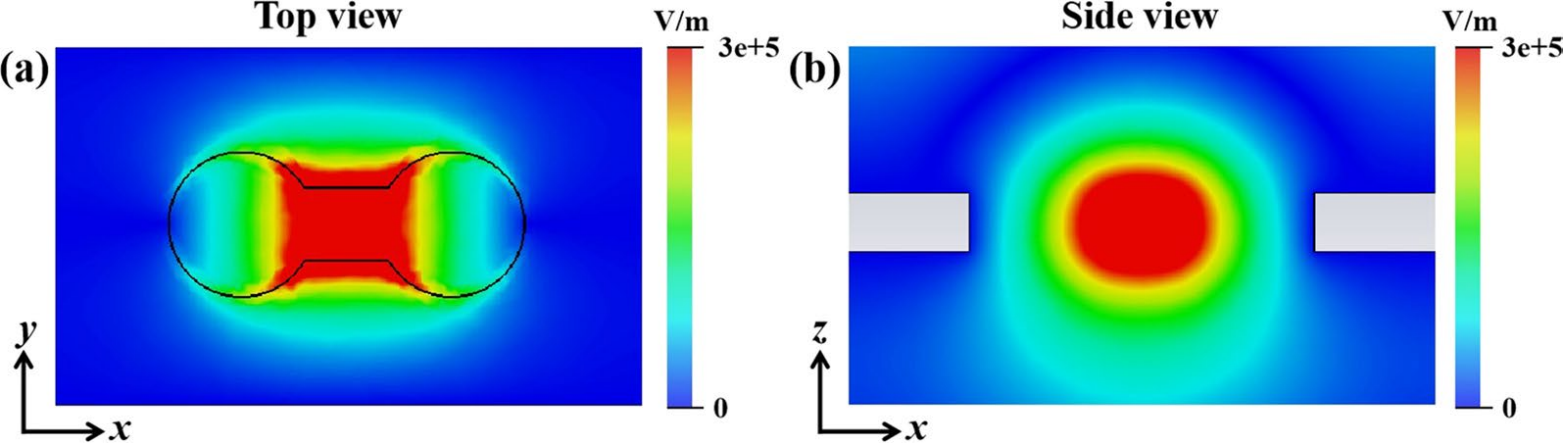
Simulated electric field distributions. **a** Top view, **b** side view (*y* = 0 μm)

To further investigate the biosensor’s sensing principle, the electric field distribution diagrams were simulated, as shown in Fig. 7. The top and side views of the simulated electric field distribution demonstrated that the electric field energy was mainly concentrated in part of the stainless-steel holes. Therefore, it is crucial to make sure that the analyte was added into the holes.

Table 1 summarized the proposed sensor’s RI sensitivity and FOM, and compared them with other reported studies [35–37]. The other THz sensors are all based on traditional photolithography processes. It can be seen that the stainless-steel metamaterial biosensor we designed had excellent sensing performance using cheap laser-drilling technology.

**Table 1:** Comparison of various terahertz sensors

Sensitivity (GHz/RIU)	Wavelength band (THz)	FOM	RI range of analyte	References
182	0.6–1.2	–	1.0–1.5	[35]
76.5	0.4–1.2	1.67	1.0–2.0	[36]
74	0.2–1.0	1.23	1.0–2.0	[37]
294.95	0.2–0.7	4.03	1.0–1.4	This paper

## Experiment

### Materials and sample preparation

Then in order to demonstrate the biosensing ability of the proposed THz mmetamaterial sensor, protein detection was performed in the experiment. BSA and PBS buffer were both purchased from Sigma-Aldrich. The BSA solution was formulated in PBS buffer (pH = 7.4).

**Fig. 8:**
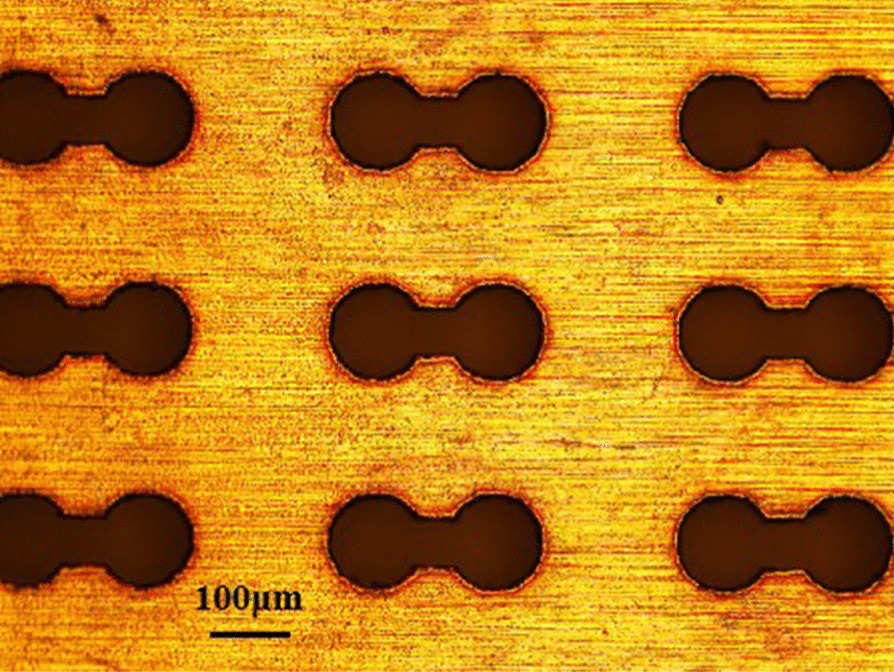
Micrograph of the fabricated biosensor sample

A microscope image of the fabricated metamaterial biosensor is shown in Fig. 8. The overall size of the metamaterial biosensor was 12 mm × 12 mm.

**Fig. 9:**
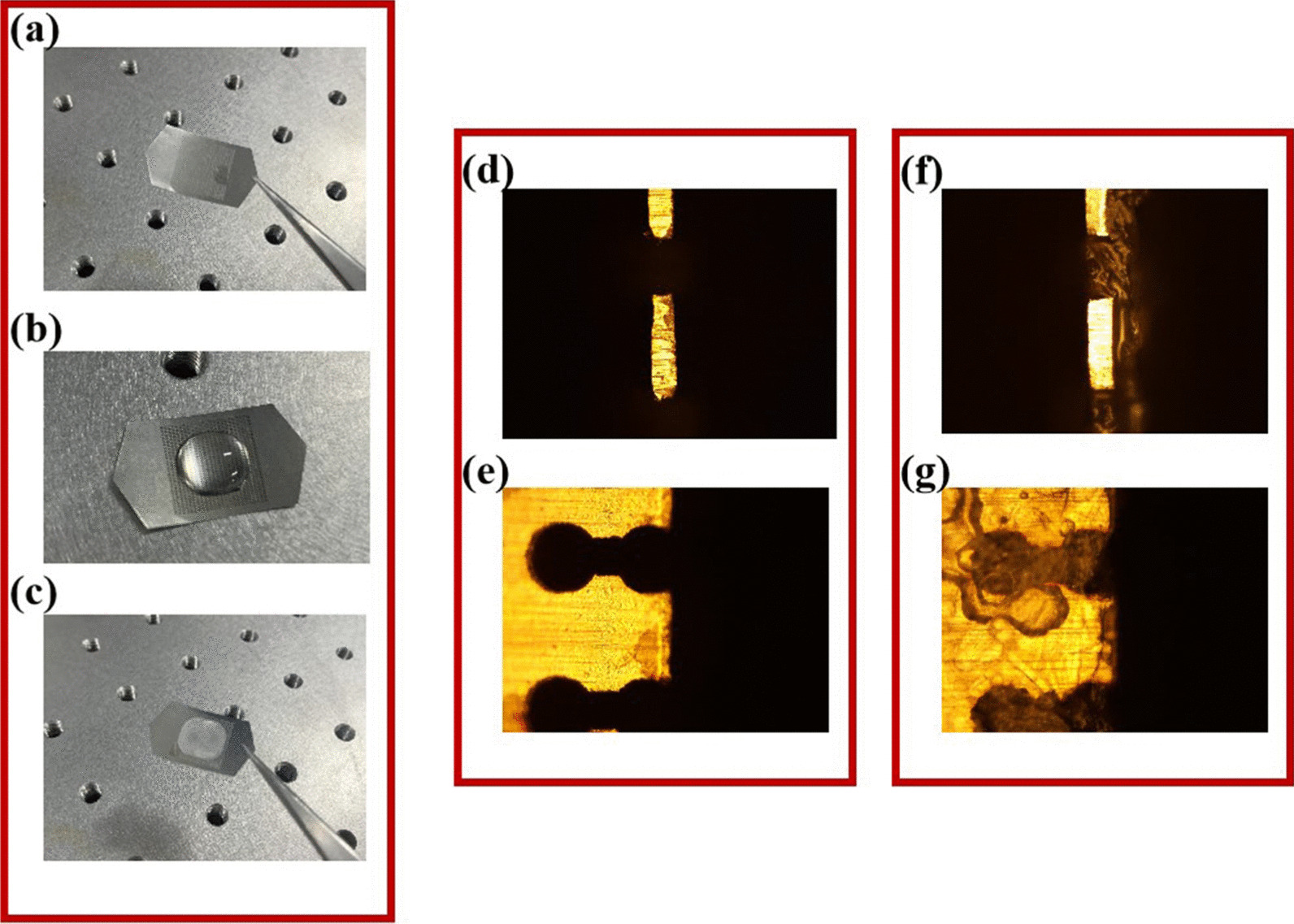
Pictures of the stainless-steel in different testing steps: the metamaterial biosensor after **a** ultrasonic cleaning and drying, **b** adding 150 μL BSA solution (0.2 mg/mL), and **c** drying; Microscope photos of the stainless-steel sheet’s **d** sidewall and **e** surface before adding BSA; Microscope photos of the stainless-steel sheet’s **f** sidewall and **g** surface after adding and drying the BSA solution (0.2 mg/mL)

The concentrations of the formulated BSA solution were 0.2 mg/mL, 0.5 mg/mL, 2.0 mg/mL, and 4.0 mg/mL. The analyte was added to the biosensor surface by liquid deposition method. Each time, 150 μL BSA solution was transferred to the biosensor surface with a pipette gun, and the biosensor was dried on a 40 ℃ heating table. When the biosensor was heated at 40 ℃, the protein film formed faster and more uniformly. Each time before changing different concentrations of BSA solution, the stainless-steel sheet was put into deionized water and vibrated in an ultrasonic vibrator to ensure that the protein film from the previous process is cleared and the surface of this mmetamaterial biosensor is clean. Figure 9 showed the pictures and the microscopy pictures of the process of adding and drying the protein. As shown in Fig. 9a, the stainless steel sheet was clean, and then as shown in Fig. 9b, the BSA solution of one concentration was added to the stainless-steel sheet’s surface, and the solution stayed on this biosensor’s surface and did not pass through the holes due to the effect of water surface tension. After heating and drying, a thin layer of the BSA film was formed as shown in Fig. 9c.

In order to observe the situation inside the stainless-steel’s holes, one side of the stainless-steel was cut off, so that one side of the holes was revealed and observed with a microscope. As shown in Fig. 9d–g, when the BSA solution was dripped and dried, a thin layer of the BSA was added into the stainless-steel’s holes, which is mainly because the diameter of the holes is much larger than the size of the BSA proteins. This proves that the analyte to be detected can enter the detection sensitive region of our biosensor, which can greatly increase the sensitivity of this metamaterial biosensor.

### Spectral measurements

**Fig. 10:**
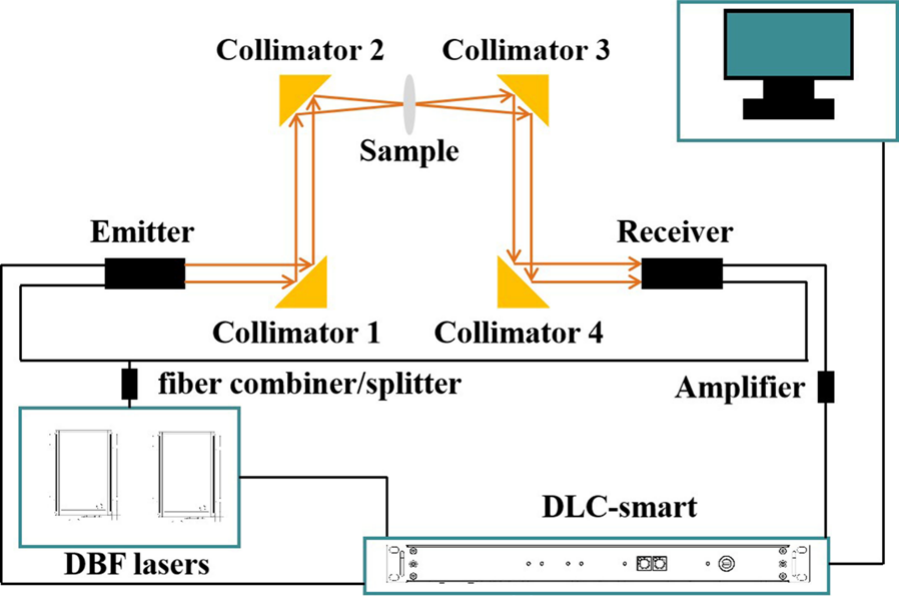
Schematic diagram of the continuous-wave THz spectrometer used in our experiments

All the spectral measurements were conducted using a continuous-wave THz spectroscopy system (TeraScan 1550, Toptica Photonics AG). The system consisted of dual-laser control (DLC) smart electronics, two distributed feedback (DFB) lasers, two fibre-coupled InGaAs photomixers, and four 90° off-axis parabolic mirrors as shown in Fig. 10. THz waves were collimated and focused on the sample through the 90° off-axis parabolic mirrors. All transmission spectra were obtained by scanning between 50 and 1220 GHz in step sizes of 40 MHz with an integration time of 10 ms operating in the fast scan mode to reduce the scanning time. The polarization of the terahertz wave was along the dumbbell ring’s opening direction.

## Experiment results and discussion

The protein detection experiments of the metamaterial biosensor were carried out with four concentrations of the BSA solutions. Throughout the experiments, each group of BSA solutions was added in order from low to high. All the measurements were repeated three times.

**Fig. 11:**
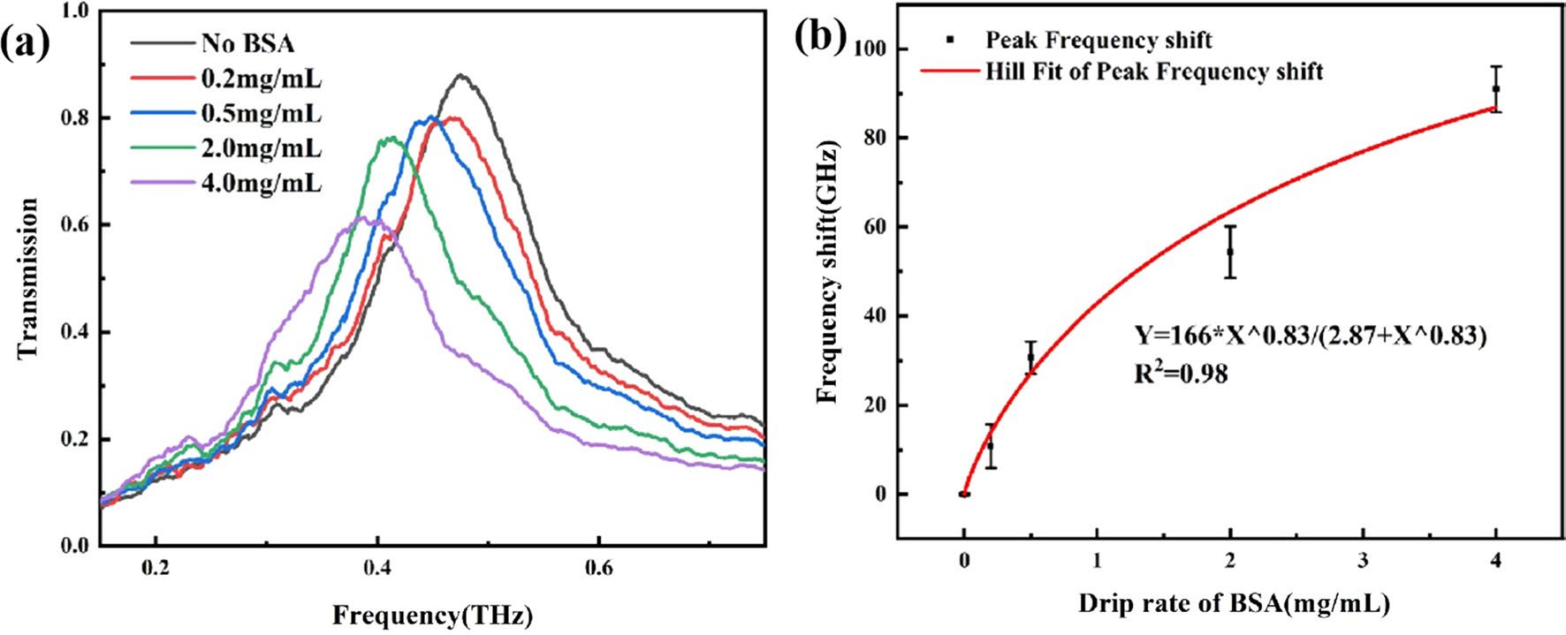
**a** Measured spectra with different BSA concentrations. **b** Hill fit of the BSA experiment

As shown in Fig. 11a, as the concentration of the BSA solutions increased, the resonance peak frequency red-shifted. This trend is consistent with the simulation results. The decrease in the intensity of the resonance was due to the absorption of the terahertz wave by the BSA protein.

The relationship between the frequency shift and the concentration of the BSA solutions is not linear, which is common in biological experiments [38, 39]. The Hill model can characterize the binding ability between our metamaterial biosensor and biomolecules. Therefore, the Hill model [40] was used to fit the experimental data, as shown in Fig. 11b. The Hill equation is described as follows:

2 $$\Delta f = \Delta f_{{\max }} \cdot \frac{{[{\text{BSA}}]^{n} }}{{\left\{ {K_{{\text{D}}} + [{\text{BSA}}]^{n} } \right\}}}$$

where the maximum peak frequency shift Δ*f*_max_ is the saturation value, [BSA] is the concentration of the BSA solution, *n* is the Hill coefficient, and *K*_D_ is the dissociation constant.

Using the fitting curve as shown in Fig. 11b, the Hill coefficient *n* was calculated to be 0.83, and the dissociation constant *K*_D_ was calculated to be 2.87 mg/mL. Furthermore, Δ*f*_max_ was approximately 166 GHz, which indicated the maximum peak frequency shift at the saturation concentration. The Hill fitting results of the resonant frequency peak confirmed the experiment’s reliability and accuracy.

The biosensor’s sensitivity S was deduced as follows [41]:

3 $$S = \frac{{\Delta f_{{\max }} }}{{\delta _{{\max }} }}$$

where Δ*f*_max_ is 166 GHz and *δ*_max_ is the BSA’s surface density. *δ*_max_ was determined by [41]:

4 $$\delta _{{\max }} = \frac{{M_{{{\text{BSA}}}} }}{{N_{{\text{A}}} \times P_{{{\text{BSA}}}}^{2} }}$$

where *M*_BSA_ = 66,430 g/mol is the estimated molecular mass of BSA [42], *N*_A_ = 6.02 × 10^23^ mol^−1^ is Avogadro’s number, and *P*_BSA_ = 6.96 nm [43] is the average length of one BSA molecule. *δ*_max_ was 2.28 ng/mm^2^ and the biosensor’s BSA detection sensitivity was 72.81 GHz/(ng/mm^2^).

*K*_D_ obtained using the Hill model showed that the dissociation constant was strongly related to the BSA, and the limit of detection (LOD) *C*_lim_ of the BSA was calculated using the following equation [44]:

5 $$C_{{\lim }} = K_{{\text{D}}} \times \frac{{S_{{\text{f}}} }}{{\Delta f_{{\max }} - S_{{\text{f}}} }}$$

where *S*_f_ is the spectral resolution of 2 GHz. Equation (5) shows that smaller dissociation constant resulted in a lower detection limit. Thus, *C*_lim_ was calculated to be 0.035 mg/mL.

Table 2 shows our biosensor’s BSA sensing performance compared with those reported studies. In the experiment, the lowest concentration of the BSA solution was 0.2 mg/mL and a frequency change of 10.8 GHz was obtained. Compared with Refs. [45–47], a relatively higher frequency change was achieved at the same BSA concentration. The Hill's formula was applied to analyze the metamaterial biosensor’s data. The calculated LOD of 0.035 mg/ml was significantly better than that in Ref. [45]. All these predict that our proposed all-metal THz metamaterial biosensor will have excellent performance in many biological and chemical applications.

**Table 2:** Comparison of various biosensors for detecting BSA

BSA detection sensitivity	Frequency shift (GHz)/lowest concentration (mg/mL)	Limit of detection (mg/mL)	Wavelength band (THz)	References
1.43 GHz/(mg/ml)	50/49.8	1.18	1.0–1.7	[45]
**–**	2/0.1	**–**	0.6–1.0	[46]
**–**	300, 400/9	**–**	0.1–1.4	[47]
72.81 GHz/(ng/mm^2^)	10.8/0.2	0.035	0.2–0.7	This work

Based on the excellent sensing performance of stainless steel biosensor, the stainless steel biosensor can be modified with specific antibody to achieve specific antigen detection in the future. And the thickness of stainless steel biosensor is only 50 μm. With the development of microfluidics technology and terahertz spectroscopy, it is hopeful to apply real-time measurement in vivo in the future.

## Conclusion

In conclusion, a terahertz biosensor based on an all-metal metamaterial was used to measure protein concentrations. The biosensor was made of stainless-steel and prepared via laser-drilling technology. The maximum RI sensitivity and FOM calculated using CST electromagnetic simulation software are 294.95 GHz/RIU and 4.03, respectively. The sample was characterized using a continuous-wave THz spectrometer. The experimental results showed that, for the BSA analyte solution, the detection sensitivity and detection limit are 72.81 GHz/(ng/mm^2^) and 0.035 mg/mL, respectively. This biosensor has advantages of small shape, high detection sensitivity, low detection limits, reusability, easy to fabricate and cost-effective. These research results are of considerable significance for future applications in biomolecular detection and disease diagnosis.

## Data Availability

All data are fully available without restriction.

## Abbreviations

THz: Terahertz
RI: Refractive index
FOM: Figure of merit
BSA: Bovine serum albumin
PET: Polyethylene terephthalate
DLC: Dual-laser control
DFB: Distributed feedback
LOD: Limit of detection
